# Analysis of factors associated with hematopoietic stem-cell retransplantation: a case-control study

**DOI:** 10.1590/1518-8345.5794.3535

**Published:** 2022-05-16

**Authors:** Isabelle Campos de Azevedo, Marcos Antonio Ferreira, Anália Andréia de Araújo Nascimento, Allyne Fortes Vitor, Elen Ferraz Teston, Oleci Pereira Frota, Viviane Euzébia Pereira Santos

**Affiliations:** 1 Universidade Federal do Rio Grande do Norte, Departamento de Enfermagem, Natal, RN, Brasil.; 2 Bolsista da Coordenação de Aperfeiçoamento de Pessoal de Nível Superior (CAPES), Brasil.; 3 Universidade Federal de Mato Grosso do Sul, Instituto Integrado de Saúde, Campo Grande, MS, Brasil.

**Keywords:** Graft Rejection, Recurrence, Hematopoietic Stem Cell Transplantation, Bone Marrow Transplantation, Survival Analysis, Analytical Epidemiology, Rejeição de Enxerto, Recidiva, Transplante de Células-Tronco Hematopoéticas, Transplante de Medula Óssea, Análise de Sobrevida, Epidemiologia Analítica, Rechazo de Injerto, Recurrencia, Trasplante de Células Madre Hematopoyéticas, Trasplante de Médula Ósea, Análisis de Supervivencia, Epidemiología Analítica

## Abstract

**Objective:**

to analyze the factors associated with the failure of Hematopoietic Stem Cell Transplantation (HSCT) in patients undergoing Hematopoietic Stem Cell Retransplantation (HSCR).

**Method:**

this study implemented a quantitative approach and was a case-control type which addressed patients undergoing HSCR. To do so, a paired sample of two controls was used for each case (2:1). The case group consisted of the medical records of all patients who underwent HSCR (28) and the control group (56) of those who underwent only one transplant. Three variables guided the pairing: gender, diagnosis and type of transplant.

**Results:**

a total of 24 (85.71%) patients in the case group were re-transplanted due to disease relapse and four (14.29%) due to graft failure. There was a statistical difference in the analysis between patients who did not use ursodeoxycholic acid, opioid analgesics and immunosuppressants. The need for HSCR among those who used these medications inappropriately was 16.12, 12.79 and 4.5 times more likely, respectively, than those who used them correctly.

**Conclusion:**

there was a difference regarding the reasons which led to the retransplantation and the analyzed subjects, and this study concluded that the predictive reason for retransplantation in the studied sample was disease relapse.

## Resumo

**Objetivo:**

analisar os fatores associados ao insucesso do Transplante de Células-Tronco Hematopoiéticas (TCTH) em pacientes submetidos ao retransplante de Células-Tronco Hematopoiéticas (RCTH).

**Método:**

estudo quantitativo do tipo caso-controle para avaliar pacientes submetidos ao RCTH. Para tanto, utilizou-se amostra pareada de dois controles para cada caso (2:1). O grupo caso foi constituído pelos prontuários de saúde com todos os pacientes que foram submetidos ao RCTH (28) e o grupo controle (56) incluiu pacientes que receberam apenas um transplante. Três variáveis nortearam o pareamento: sexo, diagnóstico e tipo de transplante.

**Resultados:**

vinte e quatro (85,71%) pacientes do grupo caso receberam retransplante devido a recidiva da doença e quatro (14.29%) devido a falha do enxerto. Uma diferença estatística foi encontrada na análise entre os pacientes que não usaram o ácido ursodesoxicólico, analgésicos opioides ou imunossupressores. A necessidade de um RCTH entre aqueles que usaram estes medicamentos de forma inapropriada foi 16,12, 12,79 e 4,5 vezes maior, respectivamente, do que entre os que as usaram corretamente.

**Conclusão:**

houve uma diferença relacionada ao motivo que levou ao retransplante e os indivíduos analisados. A conclusão é que a razão preditiva para retransplante nesta amostra foi a recidiva da doença.

## Resumen

**Objetivo:**

analizar los factores asociados con el fracaso del Trasplante de Células Madre Hematopoyéticas (TCMH) en pacientes sometidos al Retrasplante de Células Madre Hematopoyéticas (RCMH).

**Método:**

estudio cuantitativo de tipo caso-control que abordó pacientes sometidos al RCMH. Para esto, se utilizó una muestra pareada de dos controles para cada caso (2:1). El grupo caso estuvo formado por los registros médicos de todos los pacientes que fueron sometidos al RCMH (28) y el grupo control (56) por los que fueron sometidos a un solo trasplante. Tres variables guiaron el emparejamiento: género, diagnóstico y tipo de trasplante.

**Resultados:**

un total de 24 (85.71%) pacientes en el grupo caso fueron retransplantados debido a la recaída de la enfermedad y 4 (14.29%) por el fracaso del injerto. Hubo una diferencia estadística en el análisis entre los pacientes que no usaron ácido ursodesoxicólico, analgésicos opioides e inmunosupresores. La necesidad de RCMH entre los que usaron estos medicamentos de manera inapropiada se encontraba 16,12 - 12,79 y 4,5 veces más probable, respectivamente, que aquellos que los usaron correctamente.

**Conclusión:**

hubo diferencia en cuanto a las razones que llevaron al retrasplante de los sujetos analizados. Este estudio concluyó que la razón predictiva del retrasplante, en la muestra estudiada, fue la recidiva de la enfermedad.

**Highlights**

(1) First Brazilian study on hematopoietic stem cell retransplantation (HSCR).

(2) It presents evidence on the clinical profile of patients undergoing HSCR.

(3) The predictive reason for HSCR was disease relapse.

(4) The disease relapse maybe is associate with the treatment fail.

## Introduction

Hematopoietic stem cell transplantation (HSCT) is considered a treatment form for malignant hemopathies, as well as other hematopoietic, lymphatic, and immune system diseases^1^. It is characterized by intravenous hematopoietic progenitor cell (HPC) grafting in order to correct quantitative or qualitative bone marrow failures^2^.

HSTC can be classified according to its type; as autologous when the HPC are from the patient themself, as allogeneic when the cells are from donors (related or not) with compatible human leukocyte antigen (HLA), and syngeneic when the HPC are from an identical twin. The cells used for this type of transplant can be collected from bone marrow, peripheral blood or umbilical cord and placental blood^2^ ^-^ ^3^.

It is essential that the infused cells proliferate in the recipient permanently to avoid rejection to achieve HSCT success, and that the new immune system received from the donor tolerates the recipient’s tissues in order to avoid infections, Graft versus Host Disease (GVHD) and other morbidities, which can be serious and fatal. In addition, the immune system must be functioning properly and the disease stage, its complications, comorbidities at the time of transplant and healthcare throughout the process must also be considered^4^. 

For the purposes of this study, the condition in which the performed HSCT is unable to establish itself in the patient who received it and to resolve the initial bone marrow failure, and which has required retransplantation as a therapeutic condition will be considered as a failure.

HSCT failure is directly related to all phases of the procedure, being represented by graft failure or rejection and disease relapse, which occurs in about 20% of transplant patients^5^. A study carried out in Germany corroborates this statistic, as 24.89% of the 229 patients who underwent HSCT between January 2005 and December 2015 required retransplantation^6^. There are no epidemiological data from studies on HSCR carried out in Brazil, with this being the first study on the subject.

In this perspective, this constitutes an epidemiological study carried out with the objective of investigating the factors associated with HSCR which could identify possible characteristics or complications that occurred during the transplantation process and that can significantly contribute to the success or failure procedure such as GVHD, graft failure or relapse, among others^6^.

HSCT failure can culminate in the need for retransplantation and will again expose the patient to all of the procedure stages and to the morbidity and mortality risks. Hematopoietic Stem Cell Retransplantation (HSCR) can be defined as the only treatment option/form for rejection, graft failure or relapse with a chance of increasing survival or promoting remission of the disease in patients who have previously undergone HSCT^7^. The prognosis for patients with disease progression after first HSCT is dismal, and HSCR is a potentially curative option^8^.

When considering that the epidemiological profile of patients as well as the factors associated with retransplantation vary according to the underlying disease and the clinical course of treatment, the present study was anchored in the following research question: Is there an association between clinical and sociodemographic characteristics with hematopoietic stem-cell retransplantation?

This study is mainly justified by the complexity involved in the HSCT procedure^9^ and because identifying variables which can lead to the risk of an HSCR requires thorough investigation. This in turn justifies the need to carry out longitudinal studies such as this one in order to identify and analyze these intrinsic factors of the retransplantation process to produce knowledge that supports healthcare, the development or improvement of institutional care protocols and public policies for the effective functioning of transplant services.

In this context, it is expected that the results presented herein can contribute to systematizing and improving the organization and planning of the care provided to HSCT patients, in addition to fostering training of health professionals, especially nurses, with a view to capacitating them to provide specialized care regarding HSCT and its interfaces based on identifying factors which lead to HSCR and the prevention of associated complications.

Therefore, the objective of the present study is to analyze the factors associated with the failure of hematopoietic stem-cell transplants in patients submitted to HSCR. 

## Method

### Study design

This study had a quantitative approach and was of the case-control type of analytical, observational, longitudinal and individual design with a proportion of 1 case/2 controls in patients undergoing HSCT. The Strengthening the Reporting of Observational Studies in Epidemiology (STROBE) guide for observational studies was followed, as recommended by the EQUATOR network.

### Study location and period

The study was conducted in a referral hospital for high complexity care located in Natal/RN, Brazil, authorized, accredited and qualified to conduct HSCT by the Brazilian Unified Health System (*SUS*) starting in 2004. Data were collected between the months of July and August 2018.

### Participants

The sample comprised all patients with registered medical records who underwent HSCT at the service under study, between January 2008 and December 2017, for a total of 10 years of the procedure. This time frame is justified by the fact that the medical records of the years between 2004 and 2007 were not registered with the Medical and Statistics Archive Service.

The study population consisted of all medical records registered by the service, with an initial sample of 389 records of patients who underwent the HSCT procedure, regardless of the type of transplant adopted; however, five were excluded due to illegibility or incomplete information. After excluding illegible medical records or with incomplete information, the final cohort sample consisted of 384 medical records of patients undergoing HSCT. 

A total of 31 from the total number of patients underwent HSCR, characterizing them as the case group, with the record of a single transplant performed being used to compose the control group. Three cases were excluded from the sample, as it was not possible to compare them with the controls available in the population of this study; one of them with a diagnosis of essential thrombocytopenia, another of myelodysplastic syndrome and the last patient with a diagnosis of multiple myeloma, one of the most prevalent types of hematologic cancer; however, the type of transplant performed was allogeneic due to the impossibility of using the patient’s own HPC for autologous HSCT, thus totaling 28 cases. 

The sample matching sought maximum similarity between the individuals. The control group in relation to the case group was defined by comparing the variables of gender, diagnosis and type of HSCT performed. The age variable was used to provide greater similarity between the ages of cases and controls, since ages were not equal for some pairings, but approximate. 

**Figure 1:**
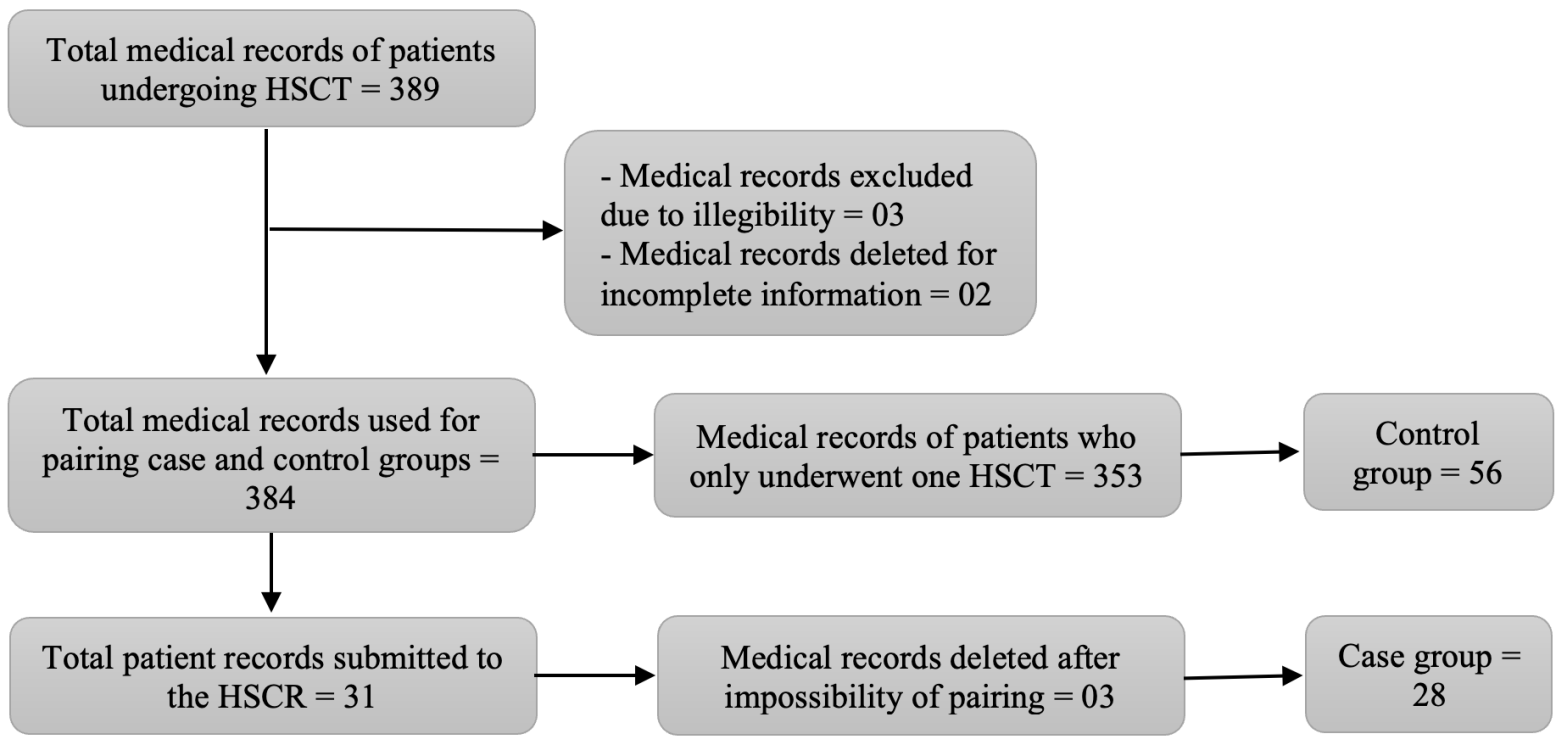
Synthesis flowchart of the selection of medical records which composed the case and control groups. Natal, RN, Brazil, 2019 (n=84)

The registry variable was used as additional information to better differentiate the medical records and minimize selection bias. It is important to highlight that the sample records of 12 children and adolescents are part of the sample, and that they were paired with each other so that the degree of similarity was met. In this context, the final sample consisted of 84 patients, 28 cases and 56 controls, the latter extracted from the 353 remaining medical records. A flowchart presented in Figure 1 was prepared for a better understanding of the selection of records by group.

The inclusion criteria for the case group were: being a patient submitted to autologous, allogeneic or syngeneic HSCR, of any age, and of both genders. Inclusion criteria for the control group were: patients submitted to only one autologous, allogeneic or syngeneic HSCT, of any age, and of both genders.

### Data collection and variables

A data collection instrument was implemented which addressed sociodemographic and clinical variables, namely: date and place of birth, place of residence, age, gender, race, education level, marital status, occupation, situation with the service transplant time, time between admission and HSCT, total follow-up time by the transplant service, diagnosis/underlying disease, type of HSCT performed, type of HSCR performed, type of cells infused, reason/indication for transplantation, toxicities presented, treatments instituted, presence of acute or chronic GVHD, survival of the transplant patient. It is worth noting that all variables were collected from medical records.

The researchers who collated the data received training in two stages; the first involved the study object and its interfaces, and the second was regarding the data collection instrument and its specificities. They were blinded to the study objectives as well as to the hypothesis to be tested as a way to minimize confusion bias in an attempt to make any association between the responses. 

### Data processing and analysis

Tables were constructed with absolute and relative frequencies by gender and total, means and standard deviations for the sample description. The chi-squared test was used to test for statistical significance, Fisher’s exact test was used to calculate the probability of association between the analyzed characteristics and the case group according to the nature of each variable, and the Mann-Whitney U test was used to compare central trends of two independent samples of equal sizes and to compare two paired or unpaired groups to verify whether or not they belong to the same population and whose requirements for applying the Student’s t-test were not met. The Kolmogorov-Smirnov test was used to verify the data normality.

Bivariate and multivariate analyzes were used to assess the association between the selected variables and estimate the magnitude of the Odds Ratio (OR), respectively. The Likelihood Ratio, Wald and Pseudo R^2^ tests (Cox and Snell = 0.240; Nagelkerke = 0.333) were used to ensure the significance of the variables in the Logistic Regression models, as well as for the hypothesis test to determine whether the independent variables in the model are significantly related to the dependent variable.

A dependent variable (hematopoietic stem cell retransplantation = Y) was adopted for the logistic regression, and the influence of one or more independent, causal or explanatory variables (X1, X2, X3,...) was verified on the dependent variable. The following equation was used for this analysis:

When analyzing the final logistic regression model, the Chi-squared statistic of the residuals (Overall Statistics) presented a value of p>0.05, meaning that none of the variables excluded from the model would significantly contribute to the model’s predictive power. It was found that the p-value > 0.05 was not statistically significant by the Hosmer-Lemeshow test, which indicates that a good fit of the final model was obtained. Therefore, it was concluded that the final adjusted model of logistic regression was adequate.

Survival calculations were performed by applying the Kaplan-Meier method with the patient’s initial entry point in the study being considered the date of the HSCT and the last event as the final point: death, abandonment, follow-up at the time of data collection and finalized treatment. The log-rank statistical method was used to compare the survival rates by variables to be listed. A significance level of 0.05 was adopted for all analyses.

### Ethical aspects 

The Research Protocol for this study was submitted to the Federal University of Rio Grande do Norte Research Ethics Committee in accordance with Resolution No. 466/12 of the National Health Council of the Ministry of Health which deals with research with human beings, for the evaluation of its ethical and methodological aspects^10^ and approved under opinion 2,596,384 and CAAE: 80927417.9.0000.5537, approved on April 18, 2018.

## Results

The age ranged between two and 55 years and the average was 34.11 years (± 14.20) (Table 1), while 51 (60.71%) participants were male, 65 (77.38%) lived in the state of Rio Grande do Norte, 41 (48.81%) were married, and 49 (58.33%) had paid employment. As the mean age was around 34 years and the age variable presented significant variability, two age groups were considered up to 35 years old and over 35 years old to better organize this information in Table 1. The age variable was considered continuous for association calculations and others.

**Table 1:** Bivariate analysis between the performance and non-performance of HSCR and independent variables (n=84). Natal, RN, Brazil, 2019

Variables	Groups				*Odds Ratio* [95%CI^*^]	*p* ^†^
	*case* (n=28)		*control* (n=56)			
	n	%	n	%		
**Gender**						
Male	17	60.71	34	60.71	1.00 [0.39; 2.53]	1.000^‡^
Female	11	39.29	22	39.29		
**Age range**						
Up to 35 years old	13	46.43	26	46.43	1.00 [0.40; 2.48]	1.000^‡^
Over 35 years old	15	53.57	30	53.57		
**Diagnosis**						
Acute Myeloid Leukemia	8	28.57	16	28.57	--	1.000^‡^
Non-Hodgkin’s lymphoma	6	21.43	12	21.43		
Multiple Myeloma	5	17.86	10	17.86		
Acute Lymphoblastic Leukemia	4	14.29	8	14.29		
Aplastic Anemia	3	10.71	6	10.71		
Hodgkin’s disease	1	3.57	2	3.57		
Chronic myeloid leukemia	1	3.57	2	3.57		
**HSCT^§^ performed**						
Allogeneic	17	60.71	34	60.71	1.00 [0.39; 2.53]	1.000^‡^
Autologous	11	39.29	22	39.29		
**GVHD^||^**						
No	24	85.71	55	98.21	9.17 [0.97; 86.38]	0.040^¶^
Yes	4	14.29	1	1.79		
Situation with RGH^**^						
Treatment finished	12	42.86	37	66.07	0.38 [0.15; 0.97]	0.042^‡^
Death	16	57.14	19	33.93		
**Follow-up until HSCT**						
Up to 15 days	17	39.53	26	60.47	1.78 [0.71; 4.49]	0.217^‡^
More than 15 days	11	26.83	30	73.17		
**Total follow-up time by the HSCT^§^ service**						
Up to 21 months	12	28.57	30	71.43	0.65 [0.26; 1.62]	0.355^‡^
More than 21 months	16	38.10	26	61.90		
**Survival**						
Up to 17.5 months	13	30.95	29	69.05	0.81 [0.32; 2.00]	0.643^‡^
More than 17.5 months	15	35.71	27	64.29		

^*^Confidence interval; ^†^p-value; ^‡^Chi-squared test; ^§^Hematopoietic Stem Cell Transplantation; ^||^Graft Versus Host Disease; ^¶^Fisher’s exact test; ^**^Rio Grande Hospital

From the total, 42 (50.00%) received care in the HSCT sector for about 21 months and 43 (51.19%; p<0.001) waited up to 15 days between the beginning of the conditioning regime until the first HSCT.

The diagnoses which culminated in the HSCR are shown in Table 1. A total of 35 (41.67%) of the studied patients died, of which 16 (57.14%) occurred among those who re-transplanted. The most prevalent causes of death were sepsis with 28 (33.34%), multiple organ failure in 14 (16.67%), and pulmonary infection in eight (9.52%). Moreover, 51 (60.71%) allogeneic HSCTs were performed, of which 17 (60.71%) were retransplants (Table 1).

Allogeneic HSCT occurred in 39 (76.47%) individuals, while the most used source of HPC was peripheral blood in 64 (85.33%) HSCTs, and the most indicated reason for retransplantation was disease relapse in 24 (85.71%). In addition, allogeneic HSCR was performed in 26 (92.86%) patients, being from relatives in 21 (75.00%) with peripheral blood HPC (75.00%). Patients who developed GVHD were 9.17 times more likely to perform HSCR.

The mean time to perform the retransplantation was 14.74 months, with the most prevalent reason for HSCT being disease relapse (24; 85.14%), and 42 (50.00%) had a survival of about 17 months.

A total of 24 (85.71%) of the patients in the case group in this study re-transplanted due to disease relapse, and four (14.29%) due to graft failure. When considering the average number of follow-up months by the transplant service (29.26; p<0.001) and the situation with the service in relation to death (OR = 0.38; CI = 95%: 0.15-0.97; p = 0.042), respectively, it is possible to understand that the longer the in-hospital care period, the greater the chances of patients being re-transplanted and of progressing to death. This is because the hospital stay in this context is directly related to severe complications, such as infection or sepsis which can lead to graft failure and disease recurrence. 

Table 2 presents the variables referring to the drugs which expose the patient to a greater chance of undergoing retransplantation. Furthermore, a statistically significant difference was found for patients who underwent HSCT and who developed genitourinary comorbidities, hematological toxicities and edema, where the chances of these patients undergoing retransplantation were 3.32, 3.22 and 2.81 times greater, respectively, when compared to patients who did not develop the mentioned comorbidity and toxicities. 

**Table 2:** Bivariate analysis of drugs administered and toxicities presented according to the groups studied (n=84). Natal, RN, Brazil, 2019

Variables	Groups				*Odds Ratio* [95%CI^*^]	*p* ^†^
	*case* (n=28)		*control* (n=56)			
	n	%	n	%		
**Drugs administered**						
**Ursodeoxycholic acid**						
Yes	27	96.43	35	62.50	16.12 [2.05-128.12]	0.001^‡^
No	1	3.57	21	37.50		
**Immunosuppressant**						
Yes	24	85.71	32	57.14	4.50 [1.38-14.69]	0.009^‡^
No	4	14.29	24	42.86		
**Toxicities presented**						
**Genitourinary**						
Yes	9	32.14	7	12.50	3.32 [1.08; 10.17]	0.031^‡^
No	19	67.86	49	87.50		
**Hematological**						
Yes	21	75.00	27	48.21	3.22 [1.18; 8.79]	0.019^‡^
No	7	25.00	29	51.79		
**Edema**						
Yes	19	67.86	24	42.86	2.81 [1.08; 7.30]	0.031^‡^
No	9	32.14	32	57.14		
**Ophthalmological**						
Yes	8	28.57	7	12.50	2.80 [0.90; 8.75]	0.070^‡^
No	20	71.43	49	87.50		
**Sepsis**						
Yes	8	28.57	8	14.29	2.40 [0.79; 7.28]	0.116^‡^
No	20	71.43	48	85.71		

^*^Confidence interval; ^†^p-value; ^‡^Chi-squared test

There was a statistical difference in the analysis between patients who did not use ursodeoxycholic acid and immunosuppressants. The chances of the need for HSCR among those who used these medications inappropriately due to non-adherence to treatment were 16.12 and 4.5 times more, respectively, than those who used these medications correctly. It should be noted that the first and the last are essential drugs used in a mandatory way in the allogeneic transplant and retransplantation protocols. Drug non-adherence related to ursodeoxycholic acid and immunosuppressants was recorded in 51 (60.71%) of the surveyed medical records.

Retransplantation was considered a dependent variable for applying the logistic regression model adopted for this study, and the groups were coded as control (0) and case (1). The dependent variable was subsequently converted into a probability ratio and then into a logarithmic based variable. The variables which showed statistical significance (p<0.05) were organized in a multiple logistic regression model, which in principle assumed a value of p<0.20 to analyze them together (Table 3).

**Table 3:** Multiple logistic regression adjusted according to the variables that presented statistical significance grouped to the outcome of retransplantation (n=84). Natal, RN, Brazil, 2019

Variables		Frequency		*Odds Ratio* [95%CI^*^]	*p* ^†^
Diagnosis					
Acute Myeloid Leukemia		24	1^‡^	--	1.000^§^
Chronic myeloid leukemia		3	1^‡^				
Acute Lymphoblastic Leukemia		12	1^‡^				
Non-Hodgkin’s lymphoma		18	1^‡^				
Hodgkin’s disease		3	1^‡^				
Multiple Myeloma		15	1^‡^				
Aplastic Anemia		9	0^||^				
**Toxicities**					
Hematological	Yes	48	1^‡^	3.22 [1.18; 8.79]	0.019^§^
	No	36	0^||^		
Edema	Yes	43	1^‡^	2.81 [1.08; 7.30]	0.031^§^
	No	41	0^||^		
**Comorbidity**					
GVHD^¶^	Yes	5	1^‡^	9.17 [0.97; 86.38]	0.040^§^
	No	79	0^||^		
**Infections**					
Genitourinary	Yes	16	1^‡^	3.32 [1.08; 10.17]	0.031^§^
	No	68	0^||^		
Sepsis	Yes	16	1^‡^	6.03 [1.386-26.205]	0.017^§^
	No	68	0^||^		
**Medicines used**					
Vitamins and supplements	Yes	67	1^‡^	2.78 [0.72;10.63]	0.124^§^
	No	17	0^||^		
Bile acid	Yes	62	1^‡^	24.32 [27.321; 261.608]	0.004^§^
	No	22	0^||^		
Antianemic	Yes	35	1^‡^	2.60 [1.02; 6.58]	0.042^§^
	No	49	0^||^		
Anti-hemorrhagic	Yes	33	1^‡^	1.95 [0.77; 4.91]	0.155^§^
	No	51	0^||^		
Anticoagulant	Yes	20	1^‡^	3.12 [0.91;7.17]	0.046^§^
	No	64	0^||^		
Loop Diuretic	Yes	55	1^‡^	2.56 [0.90;7.29]	0.074^§^
	No	29	0^||^		
Antiviral	Yes	79	1^‡^	0.164^**^	--
	No	5	0^||^		
Immunosuppressant	Yes	56	1^‡^	4.50 [1.38;14.69]	0.009^§^
	No	28	0^||^		

^*^Confidence interval; ^†^p-value; ^‡^Yes; ^§^Chi-squared test; ^||^No; ^¶^Graft Versus Host Disease; ^**^Fisher’s exact test

The adjusted model demonstrated that the heavier weight for the diagnosis was presented by the case group, so that only Aplastic Anemia did not present a statistical difference between the groups. The other tested variables were related to the chance of performing HSCR in a lower or higher ratio, according to the results shown in Table 3.

The final multiple logistic regression model showed a significance level with a p-value <0.05, with statistical evidence of association of the case group with the variables of sepsis and bile acid. Therefore, the chance of patients in this study affected by sepsis who used bile acid to undergo retransplantation was greater in relation to patients who did not develop sepsis and who did not use such medications.

It is worth mentioning that Hypothesis 1 for this study of “Clinical and sociodemographic variables are associated with the need to perform HSCR among patients submitted to HSCT”, was tested and accepted.

The cumulative survival probability was calculated from the total number of patients investigated in this study (n = 84), among which 49 (58.33%) completed the treatment and were discharged from the hospital, while 35 (41.67%) died. The zero (initial) time considered was the patient’s entry into the service in question to start treatment, and closure was characterized by treatment completion (when the patient was discharged from the hospital) or death. The minimum survival time was 6.67 months, and the maximum was 91.7 months. The mean overall survival (OS) was 26.97 months and 50% of the sample had an OS of 17.55 months. 

**Figure 2:**
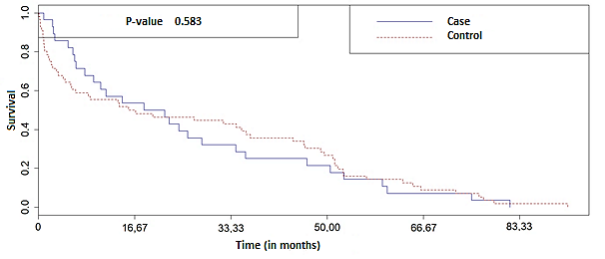
Overall survival curve between the *case* and *control* groups. Natal, RN, Brazil, 2019 (n=84)

In general, the greater the number of months, the lower the OS. Figure 2 below shows the OS curve of the total number of individuals analyzed in this study.

There was no statistically significant difference for OS between groups (Log Rank = 0.583). When determining survival by study group, it was possible to observe that the curve remained similar for both until the outcome (Figure 2), whether treatment completed or death, with a small difference for the control group in which the OS was up to 91.7 months compared to the case group, which was approximately 80 months.

## Discussion

The epidemiological profile description of the transplant patients reveals a slight predominance of males with a diagnosis of acute myeloid leukemia and the main cause of death was sepsis. The sociodemographic data in a multicenter study conducted in Europe which retrospectively followed-up patients with acute leukemia recurrence after the first HSCT are similar to those found in this study in which the mean age of the patients was 36.76 years, 58.04% were men, 21% had ± 24-month survival, 90% of patients died, and the most frequent causes were progressive disease with 136 (55.06%), sepsis in 34 (13.76%), and GVHD in 29 (11.74%)^11^.

HSCR is considered a complex and aggressive procedure, as it exposes patients to a new conditioning regime and debilitating toxicities. As in the first transplant, this phase demands specific care with a multidisciplinary team which provides care support during hospitalization and in the post-transplant with outpatient care. The treatment is long and involves risks that predispose the patient to a wide range of complications which need to be managed so that they do not threaten their survival and quality of life^12^ ^-^ ^13^.

As in post-HSCT, allogeneic post-transplantation can present some complications such as infections, toxicities, GVHD and graft failure, which comprise the main causes of long hospitalization periods and death^14^ ^-^ ^15^. Cellular and molecular mechanisms that mediate graft rejection are also triggers for developing GVHD and its control is performed by using immunosuppressants. Such complications are important problems, cause significant morbidity and mortality, and limit the transplant success and may culminate in the need for HSCR^4^.

Graft failure and disease relapse are the main reasons that lead to retransplantation mentioned in a study^16^. Factors associated with graft failure include HLA incompatibility, underlying disease, type of conditioning regimen, source of CPH employed, low HPC dose, ex vivo T cell depletion, major ABO incompatibility, grafts from female donors to male recipients and disease status at the transplantation time, infection or sepsis and non-adherence to drug therapy with ursodeoxycholic acid and immunosuppressants in the post-HSCT period^17^. The latter two mentioned were pointed out by the results of this research as factors which increase the chances of retransplantation.

Relapse is the main cause of treatment failure and its prognosis is poor^18^. Malignant cells can escape the cytotoxic lesion associated with the pre-transplant conditioning regime and the immune control created by the medullary and immune reconstitution in the post-transplant and cause disease relapse which does not respond to the chemotherapeutic drugs previously used, requiring a more cytotoxic drug arsenal^19^.

HSCT affects the intestinal microbiota, microbiome-metabolome and liver axis which can alter intestinal homeostasis and the severity of GVHD^20^. The use of ursodeoxycholic acid is considered useful in the prophylaxis and treatment of some liver conditions, especially from DECH for being one of the reasons that may lead to the need for HSCR according to the results presented by this study. A retrospective study investigated the use of ursodeoxycholic acid and found a decreased incidence of sinusoidal obstruction syndrome and other liver complications^21^.

The most commonly used bile acid in HSCT protocols is ursodeoxycholic, and its inappropriate use at a dosage lower than that prescribed had a direct relationship with retransplantation (OR = 16.12; CI = 95%: 2.05-128.12; p = 0.001) among the participants of this study with regard to protective factors against GVHD and other liver diseases. The liver is one of the target organs of GVHD, and significant changes in the serum bilirubin and alkaline phosphatase levels may indicate involvement of this condition^22^ ^-^ ^24^.

Immunosuppressant is used to protect against graft rejection and the need for HSCR, the inappropriate use of such medication in this study is linked to increased chances of HSCR (OR = 4.50; CI = 95 %; 1.38-14.69; p = 0.009), meaning that ingesting a lower dose than recommended in the medical prescription increases the chance of a new transplant. Patients who received an allogeneic transplant routinely receive immunosuppression starting with D-1 (one day before the procedure) to avoid graft rejection or failure, and for a long time after HSCT with calcineurin inhibitors and others^4^. 

However, the polypharmacy experienced by patients using various oral medications, especially in the post-HSCT outpatient phase, is a limiting factor which makes it difficult to adhere to treatment in an appropriate and continuous manner^25^ ^-^ ^26^. Without the correct intake of daily doses of medication, such as immunosuppressants and ursodeoxycholic acid, the patient can progress to graft failure, disease recurrence or GVHD, as demonstrated by the results of this study.

Such medications are prescribed to control symptoms, infections, graft rejection, and supportive medications, among others. The therapeutic scheme is complex due to the type, number and different schedule of medications, in addition to the frequent changes in prescriptions. Patients and family members assume the burden of managing polypharmacy after discharge, and adherence is essential to minimize the chances of infection, GVHD, or disease relapse^25^ ^-^ ^26^.

Although this strategy has supported great advances in modern transplant medicine, especially in controlling acute rejection, it is linked to significant problems which include the toxicity of drugs and the increased risk of opportunistic infections, among others. In addition, the immunosuppressive regimen chosen may insufficiently control graft rejection or failure, as well as GVHD. As a result, many patients have a need for retransplantation, which can be clinically challenging being limited by the low availability of donated organs, tissues or cells^4^.

Sociodemographic factors such as low education, poor socioeconomic and lack of social support status may be directly related to the infection rates, as the patient may not understand the guidelines to avoid infections or may not have sufficient economic conditions to continue with the necessary treatment. A study carried out by researchers from several countries highlighted that 66.49% of patients did not adhere to the use of immunosuppressive drugs^27^. 

Difficulty in adherence to drug therapy after HSCT can have a significant negative impact on survival, as it increases the risk of developing infections and disease relapse, which are some of the reasons with the highest odds ratios for the need for retransplantation pointed out by this study^28^.

The infections can easily progress to sepsis in immunosuppressed HSCT candidates^29^. As shown in the results of this study, patients with sepsis are more likely to have retransplantation (OR = 6.03; CI = 95%: 1.386-26.205; p = 0.017).

Patients are often unable to ingest medications orally with the advent of oropharyngeal mucositis, nausea and vomiting during hospitalization, so those who have an intravenous presentation are replaced. However, as for bile acid, the pharmaceutical presentation is only in 50 mg, 150 mg and 300 mg tablets, and 300 mg tablets are generally prescribed every 12 hours for allogeneic transplantation protocols. As previously discussed, bile acid was a protective factor against hepatic GVHD, sinusoidal obstruction syndrome, and dyslipidemia, among others which affect the liver.

Diseases that recur after a first HSCT and graft failure or rejection usually present a poor prognosis. The attempt to achieve a better outcome for patient survival with the addition of an HSCR increased overall survival by 10%. A retransplant indication is only concentrated in patients with disease persisting after the first HSCT and in the reasons mentioned above^5^.

HSCR seems a little promising approach, but there are few treatment options for relapse after the first transplant such as chemotherapy with new drugs, infusion of donor lymphocytes for allogeneic transplantation, retransplantation or a combination of them. In this context, HSCR is the treatment which favors greater OS^7^ ^,^ ^16^.

In a North American study carried out with children and adolescents with relapse of acute myeloid leukemia after a first HSCT, it was determined that survival was 88% in 100 days, 56% in 12 months, and 48% in 120 months. This fact suggests that HSCR after initial transplant failure resulted in long-term disease-free survival in 50% of children with recurrent acute myeloid leukemia^30^.

Patients undergoing HSCT or HSCR generally require complex care and nursing care through all its phases, from indication of the patient to perform the procedure until their discharge from the hospital and follow-up at the hospital. Implementing nursing care systematization (NCS) and compliance with all its stages is urgent in this context^31^ ^-^ ^33^, given that the patient can evolve quickly with complications associated with the underlying disease or toxicities.

Promoting data which support decision-making and appropriate implementations in patient care is expected to substantially contribute to improving the clinical practice of healthcare professionals, especially nurses, and make them decisive professionals in providing care to the patient in being able to recognize the possible risk factors responsible for HSCR situations.

It is important that the multidisciplinary team, especially nurses, develop care plans that support the patient’s adherence to treatment and make correct use of medication dosages, such as immunosuppressants and ursodeoxycholic acid as a way to minimize the involvement of infections, disease relapse and graft failure, as these constitute important factors that lead to retransplantation.

In addition to a personalized care plan that meets the health needs of each patient, it is essential to invest in social support, which can be represented by the figure of the family member/caregiver and the health team, especially the nursing team. Teamwork which involves the patient, the family member/caregiver and health professionals must be developed, with a focus on improving the quality of life and survival after retransplantation.

As this is a study with a secondary data source, it should be noted that there may be some biases such as loss of information due to weaknesses in records and information systems. In addition, the sample size and study design are also emphasized, because despite meeting the proposed objectives and answering the research question and the tested hypothesis, 84 medical records did not standardize the context of HSCT and HSCR in Brazil, which makes generalizations and more elaborate statistical analyzes impossible.

## Conclusion

This case-control study presented relevant scientific evidence on the performance of HSCR with regard to the clinical-epidemiological profile and performed a comparison in relation to the factors associated with the procedure and patient survival. Is it concluded that the predictive reason for retransplantation in the studied sample was disease relapse. The clinical variables of sepsis and bile acid showed a direct relationship and odds ratio with retransplantation. Sociodemographic factors are also related to HSCR, especially regarding the importance of adhering to treatment, even in the in-hospital and post-HSCT phase. 
